# Lateral entorhinal cortex is necessary for associative but not nonassociative recognition memory

**DOI:** 10.1002/hipo.22165

**Published:** 2013-08-09

**Authors:** David IG Wilson, Sakurako Watanabe, Helen Milner, James A Ainge

**Affiliations:** School of Psychology and Neuroscience, University of St AndrewsSt Mary's Quad, St Andrews, Fife, United Kingdom

**Keywords:** episodic, hippocampus, association, context, memory

## Abstract

The lateral entorhinal cortex (LEC) provides one of the two major input pathways to the hippocampus and has been suggested to process the nonspatial contextual details of episodic memory. Combined with spatial information from the medial entorhinal cortex it is hypothesised that this contextual information is used to form an integrated spatially selective, context-specific response in the hippocampus that underlies episodic memory. Recently, we reported that the LEC is required for recognition of objects that have been experienced in a specific context (Wilson et al. (2013) Hippocampus 23:352-366). Here, we sought to extend this work to assess the role of the LEC in recognition of all associative combinations of objects, places and contexts within an episode. Unlike controls, rats with excitotoxic lesions of the LEC showed no evidence of recognizing familiar combinations of object in place, place in context, or object in place and context. However, LEC lesioned rats showed normal recognition of objects and places independently from each other (nonassociative recognition). Together with our previous findings, these data suggest that the LEC is critical for associative recognition memory and may bind together information relating to objects, places, and contexts needed for episodic memory formation.

## INTRODUCTION

Episodic memory contains a rich, complex combination of different aspects of our experience including people, objects, their spatial location and the associated occasion or context. Numerous studies have shown that the hippocampus is critically involved in episodic memory in humans (Vargha-Khadem et al., 1997; Eldridge et al., 2000; Gelbard-Sagiv et al., 2008). In order to study this type of memory in animals a number of rodent models of episodic-like memory have been developed that make use of rodents' natural propensity to explore novel aspects of their environment (Eacott and Norman, 2004; Kart-Teke et al., 2006; Eacott and Easton, 2007; Good et al., 2007). These models demonstrate that as well as exploring novel objects rodents will explore familiar objects that are presented in novel places or contexts. By exploring a familiar object in a novel place within a context, rodents demonstrate an integrated memory of what has happened, where they were and on which occasion. This integrated “what-where-which occasion” memory has been suggested to be model of episodic memory in rodents (Eacott and Easton, 2010; Easton et al., 2012). Support for this suggestion has come from studies that have shown this integrated memory to be dependent on the hippocampus in rats (Eacott and Norman, 2004; Langston and Wood, 2010).

It has been suggested that episodic memory in the hippocampus is formed by combining spatial information from the medial entorhinal cortex (MEC) (Hafting et al., 2005; Barry et al., 2006; Savelli et al., 2008; Solstad et al., 2008; Lever et al., 2009) with nonspatial information from the lateral entorhinal cortex (LEC) (Hargreaves et al., 2005; Knierim et al., 2006; Kerr et al., 2007; Hayman and Jeffery, 2008; Hasselmo, 2009). This is consistent with studies showing that neurons in LEC do not show spatially modulated firing patterns (Hargreaves et al., 2005), even in cue rich environments (Yoganarasimha et al., 2011). However, LEC neurons do show some spatially tuned firing in the presence of objects (Deshmukh and Knierim, 2011; Tsao et al., 2013). Recently, we reported that increased *c-fos* expression, within LEC, was correlated with increased discrimination of novel versus familiar object-context (OC) associations and that LEC lesions impaired novel OC recognition without affecting non-associative, object recognition (Wilson et al., 2013). This role for the LEC in associating objects with the contexts in which they were experienced suggests that the LEC may be involved in associating features of an event with each other. This is consistent with recent work showing that the LEC has a role to play in associating objects with the places in which they were experienced (Van Cauter et al., 2012).

In this study we asked whether the role of the LEC is restricted to binding objects with contexts or if it has a wider role in binding together other features of events. We also wanted to ask whether LEC also has a role to play in nonassociative recognition of single aspects or components of our attended experience. In order to address these questions we assessed the effects of lesions of the LEC on associative memory for objects and places, for places and contexts, as well as associative memory for all three components (objects, places and contexts) in the “what-where-which occasion” rodent model of episodic memory. We went on to assess the effect of lesions of the LEC on nonassociative memory for objects or locations.

## METHODS

### Subjects

Male Lister Hooded rats (Harlan Olac Ltd, Bicester, UK) were housed in groups of 4 for Experiments 1 (*n* = 24; average weight at start of experiment: 408 g) and 2 (*n* = 20; average weight at start of experiment: 349 g) on a 12 h light/dark cycle. Behavioral testing was carried out 5 days a week during the light phase. The maintenance of laboratory animals and their use in scientific experiments complied with national (Animals [Scientific Procedures] Act, 1986) and international (European Communities Council Directive of 24 November 1986 [86/609/EEC]) legislation governing the maintenance of laboratory animals and their use in scientific experiments.

### Apparatus

In both experiments behavioral testing was carried out within a 67 cm square box with 40cm high walls. This box could be configured to make two different contexts. The “white” context had floor and wall inserts that were made of plain wood painted white. The “stripes” context had floor and wall inserts that were made of plain wood painted with black and white vertical stripes (5 cm width) with black plastic-coated metal mesh overlaid on the floor. The box sat 32 cm above the ground in a circular curtained arena. Prominent extra-maze cues placed on the curtains were consistently present irrespective of the contextual configuration of the box.

**Figure 1:**
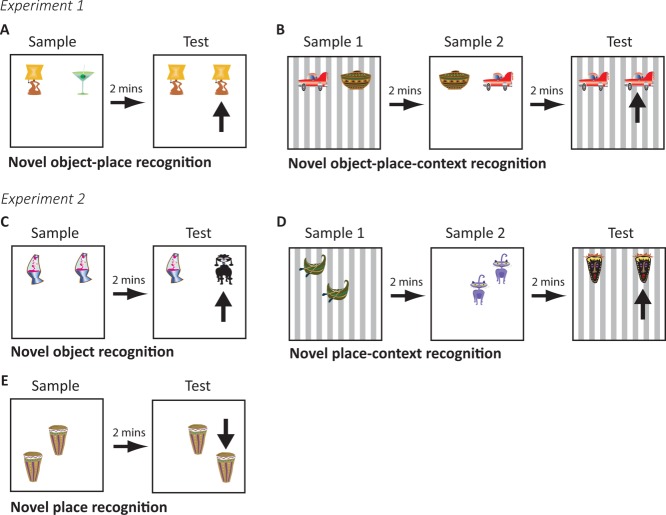
Schematic depicting the structure of a given trial within each behavioural task used in Experiments 1 (A and B) and 2 (C, D and E).The arrow in each of the test phases indicates the novel association/object/place. Different novel objects were used each day. A. Novel object-place (OP) recognition task (Experiment 1; 4 days). B. Novel object-place-context (OPC) recognition task (Experiment 1; 4 days). C. Novel object recognition task (Experiments 1 and 2; 4 days each). D. Novel place-context (PC) recognition task (Experiment 2; 4 days). E. Novel place recognition task (Experiment 2; 3 days). [Color figure can be viewed in the online issue, which is available at http://wileyonlinelibrary.com.]

The objects used were easily cleanable 3D household objects made from plastic, metal, glass, or ceramic. These were approximately the same size as a rat (in at least one dimension) and were fixed to the floor of the arena using Dual Lock (3M, St Paul, MN). For the novel object-place (OP) recognition, novel object-place-context (OPC) recognition and novel object recognition tasks, objects were positioned towards the north wall in west and east positions (Figs. 1A–C). Exploration of the objects was assessed via an overhead video recorder linked to a monitor, recorded and stored for subsequent analysis. For the novel place-context (PC) recognition and novel place recognition tasks in Experiment 2 the box was modified slightly to allow for three objects to be presented in the box. In the novel PC recognition task this was accomplished using an additional piece of Dual Lock fixed to the floor south of the other two positions and equidistant from each (Fig. 1D). For the novel place recognition task this base was rotated 180° so that the three positions for objects were now towards the south wall, creating novel positions for objects (Fig. 1E).

### Surgery

Rats in both experimental groups were initially anaesthetized using isoflurane (Abbot Laboratories, Maidenhead, UK) in an induction box. They were then placed in a stereotaxic frame (David Kopf, Tujunga CA) where anaesthesia was maintained via a facemask mounted on the incisor bar (2–3% isoflurane, 1.2 l/min O_2_). A pre-surgical analgesic Rimadyl (0.05 ml/rat; 5% w/v carprofen; Pfizer Ltd, Kent, UK) was injected subcutaneously. Following shaving the scalp, a midline incision was made and holes drilled bilaterally at stereotaxic coordinates targeting LEC: −6.5 mm from Bregma; ±4.5 mm from the midline (measured on the skull surface). Dura was cut using the bent tip of a 30 gauge needle and the pipette lowered into the brain at a 10° angle to 6.4 mm below dura. For lesioned rats (Experiment 1, *n* = 14; Experiment 2, *n* = 12) 188 nl of ibotenate (0.03 M solution in sterile phosphate buffer; Sigma-Aldrich, UK) was infused by pressure ejection from a drawn glass micropipette (tip diameter 30–40 µm) and left in situ for 5 min after infusion. Sham operated controls (Experiment 1, *n* = 7; Experiment 2, *n* = 8) underwent the identical procedure receiving only the vehicle solution (sterile phosphate buffer). Rats were given 7 days to recover from surgery before behavioral testing began.

### Behavioural Testing for Experiment 1

Following 1 week of extensive handling to habituate the rats (sham group, *n* = 7; LEC lesion group, *n* = 14) to the experimenter rats were individually habituated to contexts (4 days), to novel objects within contexts (4 days) and then tested within a novel object recognition task, as described previously (Wilson et al., 2013). Behavioral testing proceeded in the following stages:

1. Novel OP recognition task (Fig. 1A). On each day, for four days, rats were given a sample trial where they were exposed to two different novel objects in one of the contexts and allowed to explore them freely. Sample trials were terminated after 3mins or when rats had explored each object for a minimum of 15s, whichever was shorter. Rats were then removed from the box and placed in a holding cage for approximately 1min while the box was cleaned and configured for the test trial. For the test trial rats explored for 3mins within the same context as used in the sample trial except there were now two copies of one of the objects. Thus, in this test phase one of the copies had been previously presented in that location in the testing box (familiar OP association) and one of the copies was in a new location (novel OP association), which previously had held a different object. The side of presentation of the novel association in the test phase, the object used in novel or familiar association and the context used (white or stripes) were counterbalanced as much as possible within the lesion and sham groups.
2. Novel OPC recognition task (Fig. 1B; this task followed a novel OC recognition task, described previously (Wilson et al., 2013). In sample phase 1 two different novel objects were presented within context 1 (white or stripes). In sample phase 2, two different copies of these objects were presented within context 2 (stripes or white) but in opposite positions from where they were in context 1. In the test phase two further copies of one of the objects were presented within one of the contexts. Thus in the test phase one of the objects had been presented in that location and context before but not in that location within that particular context (novel OPC association), unlike the other object copy which had been seen in that location within that context before (familiar OPC association). Within lesion and sham groups we counterbalanced as much as possible the context in sample 1 (white or stripes), which object was to be in the novel or familiar association, the side for the novel association in the test phase and whether the context used in the test phase had been experienced in sample phase 1 or 2.

### Behavioral Testing for Experiment 2

Following 1 week of extensive handling with a second group of rats (sham group, *n* = 8; LEC lesion group, *n* = 12) behavioral testing proceeded in the following stages:

1. Habituation to the contexts was slightly different to Experiment 1. For the first 2 days rats were placed in the testing box and allowed to freely explore in their cage groups for 30 min and for the next 2 days they were placed individually for 10 min each. Half the rats experienced the contexts in the order white, stripes, white, stripes and the other half experienced stripes, white, stripes, white.
2. Habituation to objects in the box. Rats were placed individually in the testing box containing two different novel objects and allowed to freely explore. Across four testing days each rat experienced each context twice. Sessions lasted 10 min per day.
3. Novel object recognition task (Fig. 1C). On each day, for 4 days, rats were given a sample trial where they were exposed to two copies of a novel object in one of the contexts and allowed to explore them freely. Sample trials were terminated after 3 min or when rats had accumulated 15 s of exploration time at each object, whichever was shorter. Rats were then removed from the box and placed in a holding cage for ∼1 min while the box was cleaned and configured for the test trial. For the test trial rats explored for 3 min within the same context as used in the sample trial except there was a new copy of the object presented in the sample trial (familiar object) as well as a completely novel object. Within lesion and sham groups, the object that was novel or familiar, context (white or stripes), and the side for the novel object in the test phase were counterbalanced as much as possible.
4. Habituation to the three-object testing box. For two days rats were habituated to the box that allowed for three object locations equidistant to one another. Rats were placed in this modified box with three copies of the same novel object. Each rat experienced both contexts with half experiencing the white followed by the stripes context and the other half experiencing the stripes followed by the white context.
5. Novel PC recognition task (Fig. 1D), adapted from Easton et al. (2011). In sample phase 1 two copies of a novel object were placed in the center position and either the left or right position of context 1 (white or stripes). In sample phase 2 two copies of a second novel object were placed in context 2 (stripes or white) at the center position and the opposing left/right position to that used in sample phase 1. In the test phase two copies of a third novel object were placed at the left and right positions of either context 1 or 2. Thus one of the objects within the test phase was in a place that had been experienced in that context before (familiar PC association) while the other object was in a place where no objects had previously been experienced in that context (novel PC association). Within lesion and sham groups we counterbalanced as much as possible the context (white or stripes) and side (place) for the novel or familiar association as well as the recency with which the novel associated context had been experienced (i.e., choosing the test phase context to be the same as sample phase 1 or 2).
6. Habituation to new object locations. Three new spatial positions were created by rotating the floor insert 180° into a new fixed position. All rats experienced a single session in the box within the white context with two objects present in either the left and center, right, and center or left and right positions.
7. Novel place recognition task (Fig. 1E). On each day, for 3 days, rats experienced two copies of the same object in two locations. In the test phase one copy of the same object was placed in one of the locations used previously in the sample phase (familiar place) and another copy placed in a new location (new place). We interpreted exploration of the object in the new location in comparison to the other copy as recognition of a new place. The white context was used throughout all sample and test phases. Within lesion and sham groups we counterbalanced as much as possible the two places used in sample and test phases.

### Perfusions

Rats were humanely euthanised with i.p. injections of 200 mg/ml/kg sodium pentobarbitone (“Dolethal”, Univet, Bicester UK) and transcardially perfused with phosphate buffered saline (0.9%) followed by at least 250 ml of paraformaldehyde solution (4% made up in 0.1% phosphate buffer solution). Brains were then extracted and placed overnight in 20% sucrose solution (made up in 0.1% phosphate buffer).

### Histology

We immersed the brains in egg yolk within 24-well tissue culture plates containing paraformaldyde (40%) in the empty neighbouring wells and left them for 5 days allowing for the egg to fix onto the outside of the brains. We subsequently cut the brains into 50 µm coronal sections on a freezing microtome and mounted 1:4 sections onto slides. Sections were then stained on the slides with cresyl violet and coverslipped using DPX. Slides were viewed under a light microscope (Leitz Diaplan) at magnification x10 and x4 and lesion extent was judged by the lack of cell bodies or by cells that were shrunken and damaged.

### Behavioral Data Analysis

Rats were judged to be exploring an object when it was in close vicinity to the object with its nose directed towards it. Exploration time was not counted in moments when the rat's nose was directed away from the object even if the rat was immediately beside or even on top of the object. To check for reliability the same separate observer re-scored a subset of videos (*n* = 4) “blind” for each task (*n* = 5) and these scores were found to be consistently within 10% of the experimenter's. For each task we converted observation scores into discrimination indices (discrimination index = (time at novel - time at familiar)/(time at novel + time at familiar)) to determine the rates that the rats explored novel versus familiar objects/places/associations.

### Statistical Analysis

Separate univariate ANOVAs were made to determine the group effects of lesion (both lesion versus sham and unilateral versus bilateral lesion analyses) on the average discrimination indices and exploration rates in the test phase for each associative recognition task. One-sample *t*-tests were used to determine whether the average discrimination index over the four days for each group was different from chance (0). For each task, additional univariate ANOVAs were made to assess possible effects of lesion group on the length of time to complete the sample phase (for OP, OPC, and PC tasks repeated measures ANOVAs were made to compare group effects across sample phase 1 and 2) in order to rule out any differences between groups in sampling the objects which could have contributed to test phase effects.

## RESULTS

### Histology

#### Experiment 1

**Figure 2:**
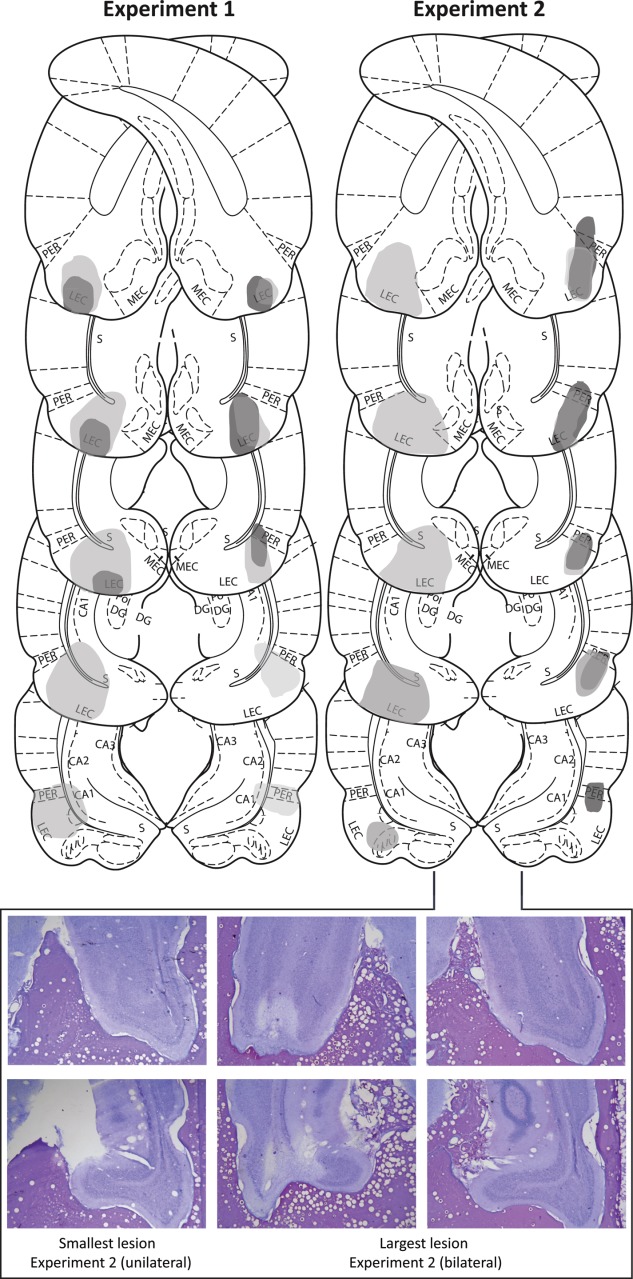
(Top) Schematic representation of lesion damage extent in Experiments 1 (left) and 2 (right) from rats with the greatest (light grey) and least (dark grey) lesion damage to LEC. Representations of coronal sections adapted from Paxinos and Watson (2011) are at −7.64 mm, −7.04 mm, −6.72 mm, −6.3 mm, −5.8 mm from Bregma, from top to bottom, respectively. (Bottom) Photographs of coronal sections of the smallest (left column; unilateral lesion also represented in schematics at −7.04 mm and −6.30 mm) and largest (centre and right columns; bilateral lesion also represented in schematics at −7.64 mm and −6.30 mm) LEC lesions from Experiment 2. Photograph examples from Experiment 1 have been reported previously (Wilson et al., 2013). [Color figure can be viewed in the online issue, which is available at http://wileyonlinelibrary.com.]

Some rats had extensive bilateral damage (*n* = 8) and others had unilateral damage (*n* = 5). One rat was excluded from all analysis due to a lack of evident lesion damage. In most rats there was some minor damage to ventral subiculum, CA1, medial entorhinal cortex, and/or perirhinal cortex although this was estimated to be <5% damage of their total area (e.g., see external damage present in the largest and smallest lesions depicted in Fig. 2). Rats with sham lesions had no lesion damage.

#### Experiment 2

Damage to the LEC and surrounding areas was similar to that in Experiment 1 with some rats having unilateral (*n* = 5) and others, bilateral damage (*n* = 5). Two rats were excluded from all analyses due to a lack of evident lesion damage. Rats with sham lesions had no lesion damage.

### Behavioral Analysis

In both experiments analyses for all behavioural tasks were initially carried out to examine whether behaviour differed between rats with bilateral and unilateral lesions. In all cases there was no significant difference between groups and so rats with unilateral and bilateral lesions were collapsed into a single group for each experiment. Additionally, for all tasks there were no effects of lesion group on the length of time it took to complete the sample phase, meaning that the groups had comparable lengths of exposure to the sampled objects.

### Experiment 1

#### Impaired associative recognition memory

**Figure 3:**
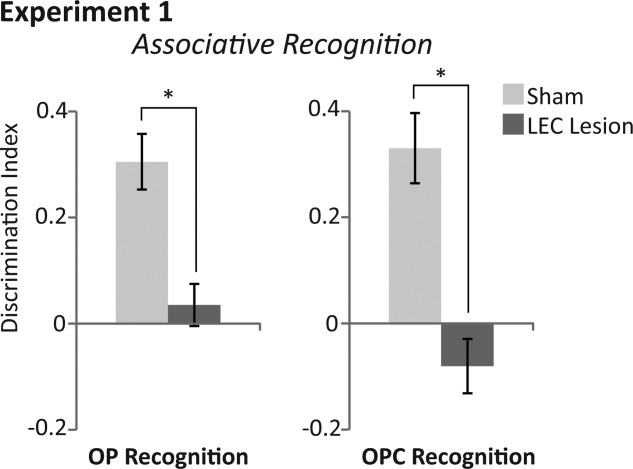
Average discrimination indices of rats with sham (light grey bars) and LEC lesions (dark grey bars) within associative recognition tasks of Experiment 1. (Left) Average discrimination indices within the object-place (OP) associative recognition task. (Right) Average discrimination indices within the object-place-context (OPC) associative recognition task. Asterisks represent a statistically significant effect (p<0.05) following ANOVA of Group (lesion versus sham) on average discrimination indices across the four days of testing.

**Figure 4:**
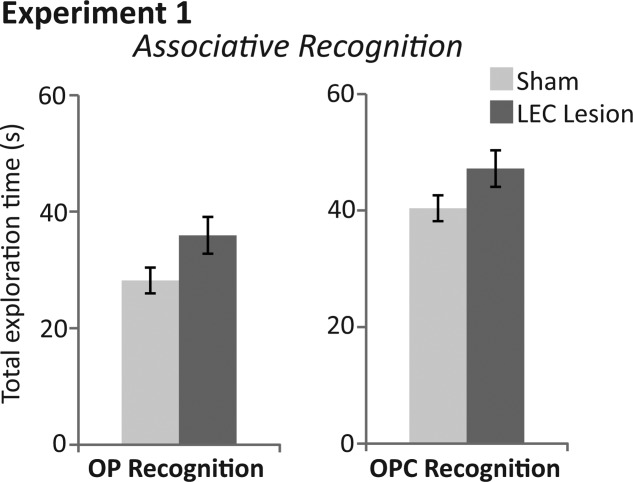
Total exploration times for rats with sham (light grey bars) and LEC lesions (dark grey bars) within associative recognition tasks of Experiment 1. (Left) Total exploration time for both objects (novel + familiar) within the test phase (3mins) of the object-place (OP) associative recognition task. (Right) Total exploration time for both objects (novel + familiar) within the test phase (3mins) of the object-place-context (OPC) associative recognition task. ANOVA revealed no significant differences in total exploration time of objects between rats with sham and LEC lesions in either task.

*Novel OP recognition*. Average discrimination indices were significantly different between sham and LEC lesion rats (*F*_(1,18)_=16.508, *P* = 0.001, partial η^2^ = 0.478; Fig. 3). Rats in the sham group had discrimination indices significantly greater than chance (*t*(6)=5.812, *P* = 0.001) demonstrating that they preferred exploring novel OP associations and therefore had remembered familiar OP associations. In contrast, rats in the LEC lesion group showed no such preference for exploring novel OP associations (*t*(12)=0.622, *P* = 0.545). There was no significant difference in the total amount of time exploring objects (time spent at novel + familiar objects) between rats in sham and LEC lesion (*F*_(1,18)_=2.830, *P* = 0.110; Fig. 4) although there was a trend towards rats in the LEC lesion group spending more time exploring objects than rats in the sham group. Mean discrimination indices for rats with unilateral (−0.018) versus bilateral lesions (0.068) were not significantly different (*F*_(1,11)_ = 1.131, *P* = 0.310).

*Novel OPC recognition*. Average discrimination indices were significantly different between sham and LEC lesion rats (*F*_(1,18)_=23.356, *P* < 0.0001, partial η^2^ = 0.565; Fig. 3). Rats in the sham group had discrimination indices significantly greater than chance (*t*(6)=4.978, *P* = 0.003) demonstrating that they preferred exploring novel OPC associations and therefore had remembered familiar OPC associations. In contrast, rats in the LEC lesion group showed no such preference for novel OPC associations (*t*(12)=−1.576, *P* = 0.141). There was no significant difference in the total amount of time exploring objects (time spent at novel + familiar objects) between rats in sham and LEC lesion (*F*_(1,18)_=0.819, *P* = 0.377; Fig. 4). Mean discrimination indices for rats with unilateral (−0.121) versus bilateral lesions (−0.055) were not significantly different (*F*_(1,11)_=0.380, *P* = 0.550).

### Experiment 2

#### Impaired associative recognition memory

**Figure 5:**
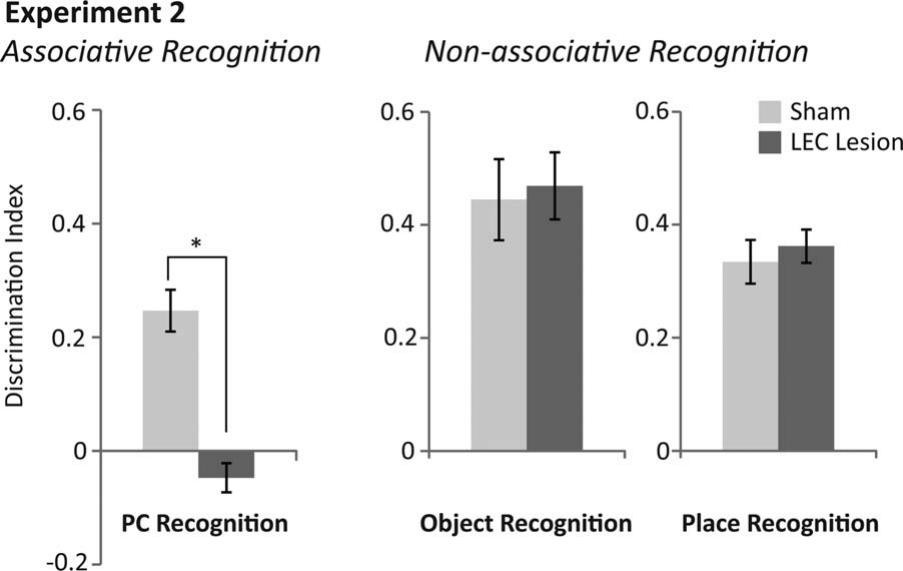
Average discrimination indices of rats with sham (light grey bars) and LEC lesions (dark grey bars) within associative and non-associative recognition tasks of Experiment 2. (Left) Average discrimination indices within the place-context (PC) associative recognition task. (Middle) Average discrimination indices within the non-associative, object recognition task. (Right) Average discrimination indices within the non-associative, place recognition task. Asterisk represents a statistically significant effect (p<0.05) following ANOVA of Group (lesion versus sham) on average discrimination indices across the four days of testing.

**Figure 6:**
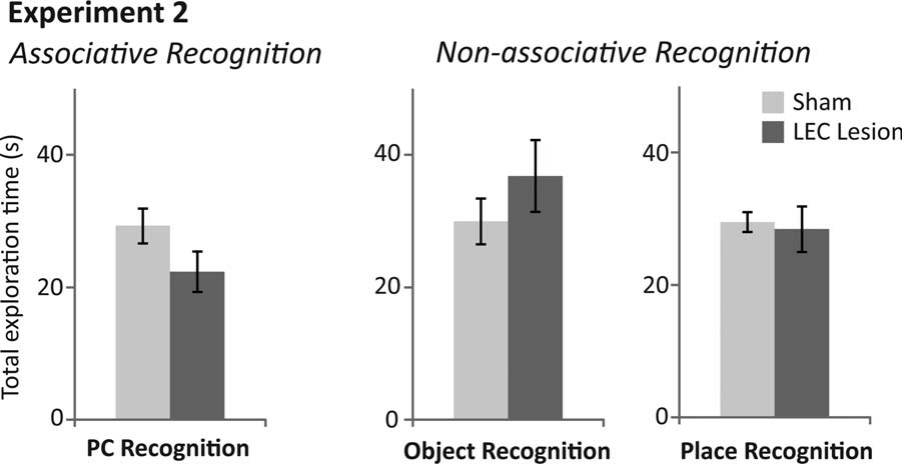
Total exploration times for rats with sham (light grey bars) and LEC lesions (dark grey bars) within associative and non-associative recognition tasks of Experiment 2. (Left) Total exploration time for both objects (novel + familiar) within the test phase (3mins) of the place-context (PC) associative recognition task. (Middle) Total exploration time for both objects (novel + familiar) within the test phase (3mins) of the non-associative, object recognition task. (Right) Total exploration time for both objects (novel + familiar) within the test phase (3mins) of the non-associative, place recognition task. ANOVA revealed no significant differences in total exploration time of objects between rats with sham and LEC lesions in any task.

*Novel PC recognition*. Average discrimination indices were significantly different between sham and LEC lesion rats (*F*_(1,16)_=39.073, *P* < 0.001, partial η^2^ = 0.709; Fig. 5). Rats in the sham group had discrimination indices significantly greater than chance (*t*(7)=6.740, *P* < 0.001) demonstrating that they preferred exploring novel PC associations and therefore had remembered familiar PC associations. In contrast, rats in the LEC lesion group showed no such preference for novel PC associations (*t*(9)= −1.871, *P* = 0.094). There was no significant difference in the total amount of time exploring objects (time spent at novel + familiar objects) between rats in sham and LEC lesion (*F*_(1,16)_=2.764, *P* = 0.116; Fig. 6). Mean discrimination indices for rats with unilateral (−0.042) versus bilateral lesions (−0.054) were not significantly different (*F*_(1,8)_=0.051, *P* = 0.827).

#### Intact nonassociative recognition memory

*Novel object recognition*. Sham and LEC lesioned rats explored the novel object more than the familiar object, demonstrating memory for the familiar object (Sham: *t*(7)=6.195, *P* < 0.001; LEC lesion: *t*(9)=7.904, *P* < 0.001; Fig. 5). ANOVA revealed no difference between discrimination indices of sham and LEC lesioned rats (*F*_(1,16)_=0.272, *P* = 0.609) or between total object exploration rates (time spent at novel + familiar objects; *F*_(1,16)_=1.229, *P* = 0.284; Fig. 6). Mean discrimination indices for rats with unilateral (0.455) versus bilateral lesions (0.484) were not significantly different (*F*_(1,8)_=0.056, *P* = 0.820).

*Novel place recognition*. Sham and LEC lesioned rats explored the novel place more than the familiar place demonstrating memory for the familiar place (Sham: *t*(7)=8.660, *P* < 0.001; LEC Lesion: *t*(9)=12.244, *P* < 0.001; Fig. 5). ANOVA revealed no difference between discrimination indices for sham and LEC lesioned rats (*F*_(1,16)_=0.334, *P* = 0.571) or between total object exploration rates (time spent at novel + familiar objects; *F*_(1,16)_=0.068, *P* = 0.797; Fig. 6). Mean discrimination indices for rats with unilateral (−0.3821) versus bilateral lesions (0.343) were not significantly different (*F*_(1,8)_=0.407, *P* = 0.541).

## DISCUSSION

LEC has been hypothesized to process the nonspatial contextual information needed to form episodic memory in the hippocampus (Hargreaves et al., 2005; Knierim et al., 2006; Kerr et al., 2007; Hayman and Jeffery, 2008; Hasselmo, 2009). In this study rats with lesions of the LEC were, unlike controls, unable to recognize any associative combinations of objects, the places in which they were experienced and the contextual features of the environment. However, they were able to recognize objects or places independently. We have previously shown that rats with LEC lesions were unable to recognize OC associations yet were able to recognise objects and contexts independently (Wilson et al., 2013). Together, these data suggest that the LEC is critical for recognition of all the possible associations between objects, places and contexts and is not required for nonassociative, independent recognition of any of these components (objects, places, or contexts).

It could be argued that these data represent a general impairment for LEC lesioned rats in complex recognition memory rather than a specific impairment in associative recognition memory between objects, places and/or contexts. Previous studies have revealed that, in a model of Alzheimer's disease, transgenic mice are specifically impaired in remembering OPC associations but are able to remember other forms of three-component associative recognition (Davis et al., 2013). This suggests that the hippocampal formation in rodents is specifically involved in episodic-like memory rather than merely being involved in any complex association, per se. Further experiments will be required to assess whether the LEC similarly has a specific role to play in episodic memory involving objects, places and/or contexts or is more generally involved in any complex, associative form of recognition memory.

One interesting issue relating to this question concerns the effects of lesions of postrhinal cortex on associative recognition memory tasks. Postrhinal cortex has also been implicated in complex object discrimination tasks (Gastelum et al., 2012; Furtak et al., 2012). However, studies using the specific tasks employed in this study have shown that rats with lesions of postrhinal cortex have been shown to have OC recognition deficits whilst having intact OPC recognition (Eacott and Norman, 2004; Norman and Eacott, 2005). This has interesting implications for models of how information is processed in the hippocampus and surrounding cortical areas. One intuitive suggestion, that is consistent with the current data, is that progressively more complex representations or associations are built up in a hierarchy as information is passed through the cortical inputs to the hippocampus culminating in episodic-like memory in the hippocampus. At the top of this hierarchy the episodic-like memory representation would be dependent on the integrity of the less complex associations made in the streams of cortical input. The fact that lesions of postrhinal cortex impair OC while leaving OPC intact argues against this suggestion. However, the current data demonstrating a deficit in all associations following LEC lesions is consistent with this hierarchical view and suggests that the associations that underlie episodic memory are formed in LEC and not in postrhinal cortex.

Previously, it has been demonstrated that whilst perirhinal and postrhinal cortices are not necessary for OPC recognition (Eacott and Norman, 2004), the hippocampus and fornix are (Eacott and Norman, 2004; Langston and Wood, 2010). The necessity for the hippocampus and LEC for OPC recognition may reflect a functional LEC-hippocampal network that processes information needed for episodic memory. Indeed, we previously reported that c-*fos* expression was increased throughout both ventral hippocampus (ventral portions of CA1, CA3, and subiculum) and the LEC during processing of objects within multiple versus single contexts (Wilson et al., 2013).

Our findings are broadly consistent with previous studies examining the roles of the structures providing the main afferent (perirhinal cortex) and efferent (hippocampus) connections with the LEC in processing the information needed to form episodic memory. The perirhinal cortex has been shown repeatedly to be necessary for object recognition (for reviews see Brown and Aggleton, 2001; Winters et al., 2008) and single neurons in perirhinal cortex decrease their firing rates as objects become more familiar (Fahy et al., 1993; Li et al., 1993; Miller et al., 1993; Brown and Xiang, 1998). Some interesting recent work has suggested that the principal role of the perirhinal cortex is in conjoining separate object components into a conjunctive representation (Bussey et al., 2002; Bartko et al., 2007; Saksida et al., 2007) and it may be involved in object-in-place recognition (Barker and Warburton, 2011) although this task has differences to the OP task used here. Importantly, in comparisons with the same tasks used here, it has been shown that the perirhinal cortex is not required for OP, OC, or OPC recognition but is required for object recognition (Ennaceur et al., 1996; Eacott and Norman, 2004; Norman and Eacott, 2005). Thus, our data suggest that object representations in perirhinal cortex are passed one synapse upstream to the LEC where they are associated with the context and place in which they were experienced.

A great number of studies have examined the role of the hippocampus in associating features of an episode. These include experiments examining the role of the hippocampus in processing context (Nadel and Willner, 1980; Maren, 2008; Rudy, 2009). A review of these studies is beyond the scope of this article but a number of findings are particularly relevant to the data presented here. The hippocampus has been shown to be necessary for complex associations of stimuli that may parallel OPC associations (Good et al., 2007; Sill and Smith, 2012). However, the hippocampus has also been implicated in associations that are possibly more basic (paralleling the OP/OC recognition tasks used here) such as learning odor-context associations for reward (Komorowski et al., 2009; Morris et al., 2013), object-context associations (Mumby, 2002; Piterkin et al., 2008) learning cue-context associations (Good et al., 1998; Honey and Good, 2000; Ainge et al., 2012), learning new odor-place (Gilbert and Kesner, 2002; Goodrich-Hunsaker et al., 2009) and object-place associations (Gaffan and Harrison, 1989; Parkinson et al., 1988; Sziklas et al., 1998; Bussey et al., 2000; Gilbert and Kesner, 2002; Crane and Milner, 2005; Lee and Solivan, 2010; Barker and Warburton, 2011) although see (Malkova and Mishkin, 2003). There are a number of factors that may affect whether the hippocampus is necessary for associative memory. These include definition of context which in some cases may involve more complex associations than others, the number of test objects and whether spatial components of the tasks are egocentric or allocentric. The data most relevant to the current studies are those examining the role of the hippocampus in the associative recognition memory tasks used here. Langston and Wood (2010) showed that the hippocampus is not necessary for OP or OC associations. This would provide support for the suggestion that object representations in perirhinal cortex are associated with places and contexts in LEC and that these representations underlie episodic memory representations in the hippocampus.

In relation to the deficits in associating objects with spatial locations reported here, our results are consistent with recent reports assessing the role of the LEC. Knierim and colleagues (Deshmukh and Knierim, 2011; Deshmukh et al., 2012) reported that when rats foraged in a testing box single neurons in LEC showed spatially specific firing patterns when objects were present. Some of these cells responded specifically to objects within the environment as well as to spatial locations where objects had previously been (see also Tsao et al., 2013). However, with relevance to our findings here, LEC neurons were only weakly spatially modulated when testing was carried out in the absence of objects (Hargreaves et al., 2005; Yoganarasimha et al., 2011) suggesting that the LEC is activated by an integration of objects and their spatial location. Similarly, Van Cauter et al. (2012) showed that the LEC may have an important role in conjoint nonspatial and spatial processing allowing for multiple associations between objects and places. Importantly, this study and others have also suggested that the LEC is not involved in spatial navigation. Unlike rats with lesions to MEC (Steffenach et al., 2005), rats with lesions to LEC (Van Cauter et al., 2012), LEC with perirhinal cortex combined (Burwell et al., 2004) or the lateral perforant path (Ferbinteanu et al., 1999), are able to successfully navigate within water maze tasks (although interference with LEC reelin signalling does impair such ability (Stranahan et al., 2011)). This is consistent with the suggestion that, unlike the MEC (Ferbinteanu et al., 1999; Van Cauter et al., 2012), the LEC is not critical for spatial navigation but is critical for associating objects and the contextual features of an environment with their spatial locations.

An important distinction in relation to spatial processing within the OPC task was made by Langston and Wood (2010). They showed that although hippocampal-lesioned rats were able to recognise OP associations, they were not able to recognise OP associations when in the test phase they were placed in the box in a new starting place. It was argued that in this revised version of the task rats must use allocentric spatial memory in order to correctly recognise OP associations whereas in the original version of the task (as used here) egocentric spatial memory is promoted as rats consistently view the objects from the same starting place and direction. Thus, it is possible that the LEC is involved in forming associations from an egocentric, rather than allocentric viewpoint (Lisman, 2007), and this would also provide an explanation for the lack of LEC processing in allocentric guided maze tasks (Ferbinteanu et al., 1999; Burwell et al., 2004; Van Cauter et al., 2012). Further research will be required to examine this hypothesis for the role of the LEC in associations between objects, places and contexts from an egocentric, but not allocentric, viewpoint.

As was the case with object-context recognition (Wilson et al., 2013), across all tasks rats with unilateral LEC lesions were equally as impaired as those with bilateral lesions. fMRI studies in humans have shown that normally functioning LEC hemispheres are highly functionally connected (Lacy and Stark, 2012). Thus, it could be the case that physical damage to one LEC hemisphere can cause a functional impairment equivalent to that from bilateral damage. Similarly, unilateral dopamine lesions can have bilateral effects on monoamine levels (Pierucci et al., 2009). Alternatively, it could be the case that there is a floor effect whereby small amounts of LEC damage cause rats to perform at chance levels whereby any subsequent amount of lesion damage cannot reveal any further cognitive impairment, as measured by these tasks. These data may have relevance to research into medial temporal lobe amnesia. Patients with damage to the medial temporal lobe exhibited deficient implicit memory for contexts composed of multiple objects in a unique spatial configuration within which a target object was associated (Chun and Phelps, 1999). These data also further our understanding of the pathology of memory-related deficits in Alzheimer's Disease since patients with Alzheimer's Disease suffer striking neural degeneration in entorhinal cortex even at the very early stage (Hyman et al., 1986; Braak and Braak, 1991; Gomez-Isla et al., 1996; Price et al., 2001; Stranahan and Mattson, 2010) and this is particularly prevalent within caudal, lateral and intermediate subfields (Hyman et al., 1986; Mikkonen et al., 1999).

## References

[b1] Ainge JA, Tamosiunaite M, Wörgötter F, Dudchenko PA (2012). Hippocampal place cells encode intended destination, and not a discriminative stimulus, in a conditional T-maze task. Hippocampus.

[b2] Barker GRI, Warburton EC (2011). When is the hippocampus involved in recognition memory?. J Neurosci.

[b3] Barry C, Lever C, Hayman R, Hartley T, Burton S, O'Keefe J, Jeffery K, Burgess N (2006). The boundary vector cell model of place cell firing and spatial memory. Rev Neurosci.

[b4] Bartko SJ, Winters BD, Cowell RA, Saksida LM, Bussey TJ (2007). Perirhinal cortex resolves feature ambiguity in configural object recognition and perceptual oddity tasks. Learn Memory.

[b5] Braak H, Braak E (1991). Alzheimer's disease affects limbic nuclei of the thalamus. Acta Neuropathol.

[b6] Brown MW, Aggleton JP (2001). Recognition memory: What are the roles of the perirhinal cortex and hippocampus?. Nat Rev Neurosci.

[b7] Brown MW, Xiang JZ (1998). Recognition memory: Neuronal substrates of the judgement of prior occurrence. Prog Neurobiol.

[b8] Burwell RD, Saddoris MP, Bucci DJ, Wiig KA (2004). Corticohippocampal contributions to spatial and contextual learning. J Neurosci.

[b9] Bussey TJ, Duck J, Muir JL, Aggleton JP (2000). Distinct patterns of behavioural impairments resulting from fornix transection or neurotoxic lesions of the perirhinal and postrhinal cortices in the rat. Behav Brain Res.

[b10] Bussey TJ, Saksida LM, Murray EA (2002). Perirhinal cortex resolves feature ambiguity in complex visual discriminations. Eur J Neurosci.

[b11] Chun MM, Phelps EA (1999). Memory deficits for implicit contextual information in amnesic subjects with hippocampal damage. Nat Neurosci.

[b12] Crane J, Milner B (2005). What went where? Impaired object-location learning in patients with right hippocampal lesions. Hippocampus.

[b13] Davis KE, Easton A, Eacott MJ, Gigg J (2013). Episodic-like memory for what-where-which occasion is selectively impaired in the 3xTgAD mouse model of Alzheimer's disease. J Alzheimers Dis.

[b14] Deshmukh SS, Johnson JL, Knierim JJ (2012). Perirhinal cortex represents nonspatial, but not spatial, information in rats foraging in the presence of objects: Comparison with lateral entorhinal cortex. Hippocampus.

[b15] Deshmukh SS, Knierim JJ (2011). Representation of non-spatial and spatial information in the lateral entorhinal cortex. Frontiers Behav Neurosci.

[b16] Eacott MJ, Easton A (2007). On familiarity and recall of events by rats. Hippocampus.

[b17] Eacott MJ, Easton A (2010). Episodic memory in animals: Remembering which occasion. Neuropsychologia.

[b18] Eacott MJ, Norman G (2004). Integrated memory for object, place, and context in rats: A possible model of episodic-like memory?. J Neurosci.

[b19] Easton A, Webster LAD, Eacott MJ (2012). The episodic nature of episodic-like memories. Learn Memory.

[b20] Eldridge LL, Knowlton BJ, Furmanski CS, Bookheimer SY, Engel SA (2000). Remembering episodes: A selective role for the hippocampus during retrieval. Nat Neurosci.

[b21] Ennaceur A, Neave N, Aggleton JP (1996). Neurotoxic lesions of the perirhinal cortex do not mimic the behavioural effects of fornix transection in the rat. Behav Brain Res.

[b22] Fahy FL, Riches IP, Brown MW (1993). Neuronal activity related to visual recognition memory: Long-term memory and the encoding of recency and familiarity information in the primate anterior and medial inferior temporal and rhinal cortex. Experimental Brain Research. Experimentelle Hirnforschung. Experimentation Cerebrale.

[b23] Ferbinteanu J, Holsinger RM, McDonald RJ (1999). Lesions of the medial or lateral perforant path have different effects on hippocampal contributions to place learning and on fear conditioning to context. Behav Brain Res.

[b24] Furtak SC, Ahmed OJ, Burwell RD (2012). Single neuron activity and theta modulation in postrhinal cortex during visual object discrimination. Neuron.

[b25] Gaffan D, Harrison S (1989). A comparison of the effects of fornix transection and sulcus principalis ablation upon spatial learning by monkeys. Behav Brain Res.

[b26] Gastelum ED, Guilhardi P, Burwell RD (2012). The effects of combined perirhinal and postrhinal damage on complex discrimination tasks. Hippocampus.

[b27] Gelbard-Sagiv H, Mukamel R, Harel M, Malach R, Fried I (2008). Internally generated reactivation of single neurons in human hippocampus during free recall. Science.

[b28] Gilbert PE, Kesner RP (2002). Role of the rodent hippocampus in paired-associate learning involving associations between a stimulus and a spatial location. Behav Neurosci.

[b29] Gomez-Isla T, Price JL, McKeel DW, Morris JC, Growdon JH, Hyman BT (1996). Profound loss of layer II entorhinal cortex neurons occurs in very mild Alzheimer's disease. J Neurosci.

[b30] Good M, de_Hoz L, Morris RG (1998). Contingent versus incidental context processing during conditioning: Dissociation after excitotoxic hippocampal plus dentate gyrus lesions. Hippocampus.

[b31] Good MA, Barnes P, Staal V, McGregor A, Honey RC (2007). Context- but not familiarity-dependent forms of object recognition are impaired following excitotoxic hippocampal lesions in rats. Behav Neurosci.

[b32] Goodrich-Hunsaker NJ, Gilbert PE, Hopkins RO (2009). The role of the human hippocampus in odor-place associative memory. Chem Senses.

[b33] Hafting T, Fyhn M, Molden S, Moser MB, Moser EI (2005). Microstructure of a spatial map in the entorhinal cortex. Nature.

[b34] Hargreaves EL, Rao G, Lee I, Knierim JJ (2005). Major dissociation between medial and lateral entorhinal input to dorsal hippocampus. Science.

[b35] Hasselmo ME (2009). A model of episodic memory: Mental time travel along encoded trajectories using grid cells. Neurobiol Learn Memory.

[b36] Hayman RM, Jeffery KJ (2008). How heterogeneous place cell responding arises from homogeneous grids—A contextual gating hypothesis. Hippocampus.

[b37] Honey RC, Good M (2000). Associative components of recognition memory. Current Opin Neurobiol.

[b38] Hyman BT, Van Hoesen GW, Kromer LJ, Damasio AR (1986). Perforant pathway changes and the memory impairment of Alzheimer's disease. Ann Neurol.

[b39] Kart-Teke E, De Souza Silva MA, Huston JP, Dere E (2006). Wistar rats show episodic-like memory for unique experiences. Neurobiol Learn Memory.

[b40] Kerr KM, Agster KL, Furtak SC, Burwell RD (2007). Functional neuroanatomy of the parahippocampal region: The lateral and medial entorhinal areas. Hippocampus.

[b41] Knierim JJ, Hamilton DA (2011). Framing spatial cognition: Neural representations of proximal and distal frames of reference and their roles in navigation. Physiol Rev.

[b42] Knierim JJ, Lee I, Hargreaves EL (2006). Hippocampal place cells: Parallel input streams, #subregional processing, and implications for episodic memory. Hippocampus.

[b43] Komorowski RW, Manns JR, Eichenbaum H (2009). Robust conjunctive item-place coding by hippocampal neurons parallels learning what happens where. J Neurosci.

[b44] Lacy JW, Stark CE (2012). Intrinsic functional connectivity of the human medial temporal lobe suggests a distinction between adjacent MTL cortices and hippocampus. Hippocampus.

[b45] Langston RF, Wood ER (2010). Associative recognition and the hippocampus: Differential effects of hippocampal lesions on object-place, object-context and object-place-context memory. Hippocampus.

[b46] Lee I, Solivan F (2010). Dentate gyrus is necessary for disambiguating similar object-place representations. Learn Mem.

[b47] Lever C, Burton S, Jeewajee A, O'Keefe J, Burgess N (2009). Boundary vector cells in the subiculum of the hippocampal formation. J Neurosci.

[b48] Li L, Miller EK, Desimone R (1993). The representation of stimulus familiarity in anterior inferior temporal cortex. J Neurophysiol.

[b49] Lisman JE (2007). Role of the dual entorhinal inputs to hippocampus: A hypothesis based on cue/action (non-self/self) couplets. Prog Brain Res.

[b50] Malkova L, Mishkin M (2003). One-trial memory for object-place associations after separate lesions of hippocampus and posterior parahippocampal region in the monkey. J Neurosci.

[b51] Maren S (2008). Pavlovian fear conditioning as a behavioral assay for hippocampus and amygdala function: cautions and caveats. Eur J Neurosci.

[b52] Mikkonen M, Alafuzoff I, Tapiola T, Soininen H, Miettinen R (1999). Subfield- and layer-specific changes in parvalbumin, calretinin and calbindin-D28K immunoreactivity in the entorhinal cortex in Alzheimer's disease. Neuroscience.

[b53] Miller EK, Li L, Desimone R (1993). Activity of neurons in anterior inferior temporal cortex during a short-term memory task. J Neurosci.

[b54] Monaco JD, Knierim JJ, Zhang K (2011). Sensory feedback, error correction, and remapping in a multiple oscillator model of place-cell activity. Front Comput Neurosci.

[b55] Morris AM, Weeden CS, Churchwell JC, Kesner RP (2013). The role of the dentate gyrus in the formation of contextual representations. Hippocampus.

[b56] Mumby DG (2002). Hippocampal damage and exploratory preferences in rats: Memory for objects, places, and contexts. Learn Memory.

[b57] Nadel L, Willner J (1980). Context and conditioning—A place for space. Physiol Psychol.

[b58] Norman G, Eacott MJ (2005). Dissociable effects of lesions to the perirhinal cortex and the postrhinal cortex on memory for context and objects in rats. Behav Neurosci.

[b59] Parkinson JK, Murray EA, Mishkin M (1988). A selective mnemonic role for the hippocampus in monkeys: memory for the location of objects. J Neurosci.

[b60] Pierucci M, Di Matteo V, Benigno A, Crescimanno G, Esposito E, Di Giovanni G (2009). The unilateral nigral lesion induces dramatic bilateral modification on rat brain monoamine neurochemistry. Annals NY Acad Sci.

[b61] Piterkin P, Cole E, Cossette MP, Gaskin S, Mumby DG (2008). A limited role for the hippocampus in the modulation of novel-object preference by contextual cues. Learn Memory.

[b62] Price JL, Ko AI, Wade MJ, Tsou SK, McKeel DW, Morris JC (2001). Neuron number in the entorhinal cortex and CA1 in preclinical Alzheimer disease. Arch Neurol.

[b63] Rudy JW (2009). Context representations, context functions, and the parahippocampal-hippocampal system. Learn Mem.

[b64] Saksida LM, Bussey TJ, Buckmaster CA, Murray EA (2007). Impairment and facilitation of transverse patterning after lesions of the perirhinal cortex and hippocampus, respectively. Cerebral Cortex.

[b65] Savelli F, Yoganarasimha D, Knierim JJ (2008). Influence of boundary removal on the spatial representations of the medial entorhinal cortex. Hippocampus.

[b66] Sill OC, Smith DM (2012). A comparison of the effects of temporary hippocampal lesions on single and dual context versions of the olfactory sequence memory task. Behav Neurosci.

[b67] Solstad T, Boccara CN, Kropff E, Moser MB, Moser EI (2008). Representation of geometric borders in the entorhinal cortex. Science.

[b68] Steffenach H-A, Witter M, Moser M-B, Moser EI (2005). Spatial memory in the rat requires the dorsolateral band of the entorhinal cortex. Neuron.

[b69] Stranahan AM, Mattson MP (2010). Selective vulnerability of neurons in layer II of the entorhinal cortex during aging and Alzheimer's disease. Neural Plast.

[b70] Stranahan AM, Salas-Vega S, Jiam NT, Gallagher M (2011). Interference with reelin signaling in the lateral entorhinal cortex impairs spatial memory. Neurobiol Learn Memory.

[b71] Sziklas V, Lebel S, Petrides M (1998). Conditional associative learning and the hippocampal system. Hippocampus.

[b72] Tsao A, Moser MB, Moser EI (2013). Traces of experience in the lateral entorhinal cortex. Current Biology.

[b73] Van Cauter T, Camon J, Alvernhe A, Elduayen C, Sargolini F, Save E (2012). Distinct roles of medial and lateral entorhinal cortex in spatial cognition. Cerebral Cortex.

[b74] Vargha-Khadem F, Gadian DG, Watkins KE, Connelly A, Van-Paesschen W, Mishkin M (1997). Differential effects of early hippocampal pathology on episodic and semantic memory. Science.

[b75] Wilson DI, Langston RF, Schlesiger MI, Wagner M, Watanabe S, Ainge JA (2013). Lateral entorhinal cortex is critical for novel object-context recognition. Hippocampus.

[b76] Winters BD, Saksida LM, Bussey TJ (2008). Object recognition memory: Neurobiological mechanisms of encoding, consolidation and retrieval. Neurosci Biobehav Rev.

[b77] Yoganarasimha D, Rao G, Knierim JJ (2011). Lateral entorhinal neurons are not spatially selective in cue-rich environments. Hippocampus.

